# Functionalization of Fast-Charging Hard Carbon Anode for Ah-Level Li-Ion Pouch Batteries

**DOI:** 10.34133/research.1238

**Published:** 2026-04-07

**Authors:** Yongpeng Cui, Kaian Yang, Zhijian Qiu, Rumeng Zheng, Xiang Li, Lingmei Wang, Yajun Wang, Yao Wang, Wen Li, Weiwei Pang, Yongfeng Li, Wei Xing

**Affiliations:** ^1^ College of New Energy and Materials, State Key Laboratory of Heavy Oil Processing, China University of Petroleum (Beijing), Beijing 102249, China.; ^2^ School of Materials Science and Engineering, State Key Laboratory of Heavy Oil Processing, China University of Petroleum (East China), Qingdao 266580, China.; ^3^ College of Science, State Key Laboratory of Heavy Oil Processing, China University of Petroleum (Beijing), Beijing 102249, China.; ^4^ Weifang University of Science and Technology, Shandong Province Engineering Research Center for Future Industries of Key Materials in Optoelectronics, Weifang 262700, China.; ^5^ Shandong Zhaowen New Energy Technology Co. Ltd., Weifang 262100, China.; ^6^ Petrochemical Research Institute, PetroChina Company Limited, Beijing 102206, China.

## Abstract

The development of fast-charging anode materials is crucial for overcoming the sluggish reaction kinetics of conventional graphite electrodes in lithium-ion batteries. This work presents a kilogram-scale producible hard carbon/graphene flake (HC-GF) composite anode engineered based on an “electron Velcro” conductive network design. In this architecture, highly conductive GFs are intimately integrated with the HC matrix, creating multidimensional electron/ion transport channels while effectively minimizing interfacial resistance. The resulting HC-GF anode exhibits exceptional electrochemical performance, delivering a reversible capacity of 506.4 mAh g^−1^ and maintaining 223.8 mAh g^−1^ at a 10 C rate (corresponding to 6-min fast charging). Remarkably, when blended with graphite at a 20% ratio in a 5 Ah LFP//graphite + 20% (HC-GF) pouch cell, this minimal doping level substantially enhances multiple performance metrics, particularly fast-charging capability and cycling stability. The pouch cell retains 3.39 Ah after 600 cycles at 3 C, corresponding to an impressive capacity retention of 94.6%. This study provides a valuable material strategy for advancing fast-charging lithium-ion battery technologies.

## Introduction

Lithium-ion batteries (LIBs) have emerged as the dominant energy storage technology for electric vehicles (EVs), owing to their superior characteristics including high energy density, extended cycle life, and absence of memory effect [1,2]. However, the accelerating adoption of electric mobility imposes stringent demands on the fast-charging capabilities of LIBs [1–3]. While refueling a conventional internal combustion engine vehicle typically requires under 5 min, charging a mainstream EV to 80% state of charge often necessitates 30 min or longer. This obvious disparity in energy replenishment time presents a critical bottleneck, severely compromising user convenience and hindering broader EV acceptance [4,5]. Consequently, mitigating “charging anxiety” through the development of advanced fast-charging technologies has become an urgent priority for the sustainable proliferation of electric transportation. Current research focuses on overcoming intrinsic limitations in battery materials and electrochemistry to enable rapid charging without compromising safety, longevity, or energy density [4,6–8].

The limited fast-charging capability of LIBs constitutes a major bottleneck contributing to charging anxiety in EVs. Commercially, LIB anodes are predominantly graphite-based, yet this material is constrained by inherent shortcomings, including a highly ordered microcrystalline structure and sluggish Li^+^ diffusion kinetics (10^−12^ to 10^−10^ cm^2^ s^−1^). Under fast-charging conditions in particular, graphite anodes are prone to lithium dendrite formation, resulting in rapid capacity fade and serious safety concerns [9–11]. Consequently, with the growing demand for higher energy and power density, conventional graphite-based anodes are increasingly inadequate. Recent studies have further demonstrated that hard carbon’s (HC) expanded interlayer spacing and disordered structure are key to overcoming the kinetic limitations of graphite anodes under high-rate conditions [12–15]. HC has emerged as a key anode material to overcome the ultra-fast charging limitations of LIBs, owing to its intrinsically non-graphitizable structure [15–19]. Its microstructure consists of short-range ordered graphite-like microdomains and randomly oriented, open-edged crystallites. This distinctive architecture provides multidimensional transport pathways for Li ions, enabling significantly faster diffusion kinetics (10^−10^ to 10^−8^ cm^2^ s^−1^). Leveraging these structural advantages, HC anodes enable rapid charging while preserving high capacity and long-term cycling stability, thereby overcoming the fundamental kinetic limitations of graphite-based anodes [20,21].

Herein, we report an ultra-fast-charging hard carbon/graphene flake (HC-GF) composite anode for LIBs, designed around the concept of an “electronic Velcro” architecture. This unique structure creates multidimensional pathways that enable rapid lithium-ion diffusion, leading to exceptional electrochemical performance. The HC-GF anode delivers a high reversible capacity of 506.4 mAh g^−1^, surpassing the theoretical limit of commercial graphite, and achieves a capacity of 223.8 mAh g^−1^ in just 6 min (at 10 C). Bridging the gap between laboratory research and practical application, we have successfully scaled up the synthesis of HC-GF to the kilogram level. When blended with conventional graphite, the composite significantly enhances both fast-charging capability and cycling stability. A LiFePO₄//graphite + 20% HC-GF pouch cell demonstrates a maximum capacity of 5.2 Ah, underscoring its strong potential for real-world deployment.

## Results and Discussion

**Fig. 1.**
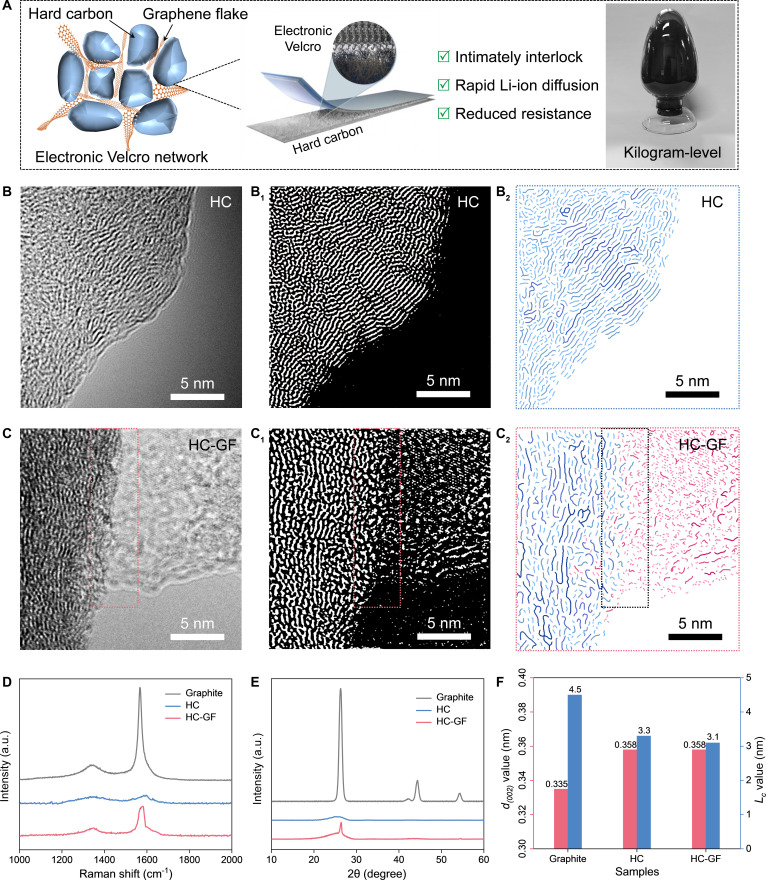
(A) Schematic illustration of the HC-GF structure and the integration strategy with electronic Velcro and photograph of the kilogram-scale sample. (B) HRTEM images and binary images of HC: (B1) HRTEM image, (B2) corresponding binary image. (C) HRTEM images and binary images of HC-GF: (C1) HRTEM image, (C2) corresponding binary image. (D) XRD patterns and (E) Raman spectra of graphite, HC, and HC-GF. (F) *d_(002)_* interlayer spacing and *L_c_* values.

Inspired by the mechanical adhesion of Velcro, we developed an “electronic Velcro” strategy to engineer the interface between HC and the conductive network. As illustrated in Fig. 1A, this architecture creates an interlocking effect between HC and GFs, ensuring structural integrity while establishing continuous electron/ion transport pathways. This design minimizes interfacial contact resistance and accelerates Li^+^ diffusion kinetics, overcoming the sluggish charge transfer typical of conventional anodes. Beyond structural design, we also achieved kilogram-scale synthesis of the HC-GF composite.

To determine the microstructure of the material, inverse fast Fourier transform is conducted on the high-resolution transmission electron microscopy (HRTEM) image. The ArcGIS software is used to extract the stripe structure in the carbon skeleton to obtain a binarized image [22–24]. As illustrated in Fig. 1B and C and Figs. S1 and S2, the HC material displays a distinctive microcrystalline structure, featuring short-range ordered graphene-like domains dispersed within a long-range disordered amorphous carbon matrix [25,26]. Based on the intensity profile for the line scan across the lattice fringes, the local interlayer spacing of HC material can reach 0.358 nm. Notably, the interfacial contact between HC and GF is clearly discernible in the HC-GF composite. Through binarization processing of HRTEM images, HC-GF can be distinctly separated into 2 parts and visualized using different color assignments, where blue represents HC material and red denotes GF material. Robust interfacial contact between HC and GF creates highly conductive pathways for electron transfer, effectively reducing internal resistance and enhancing electrode kinetics [27–29].

The phase and defect structure of all the materials are characterized by x-ray diffraction (XRD) and Raman data (Fig. 1D to F). As shown in the XRD patterns (Fig. 1D and Fig. S3), both commercial graphite and GF exhibit sharp (002) crystal plane characteristic peaks, indicating perfect crystal structures. In contrast, the HC material shows a significantly broadened (002) characteristic peak due to its long-range disordered microcrystalline structure. Significantly, the XRD profile of the HC-GF composite can be clearly indexed to the microcrystalline structures of both HC and GF, confirming their physical combination [30,31]. The Raman curve also shows the same result. HC-GF exhibits a combination of graphite-structured carbon and amorphous-structured carbon (Fig. 1E and Fig. S4). Figure 1F provides detailed structural data for the graphite, HC, and HC-GF materials. The average graphitic interlayer spacing for HC materials is about 0.358 nm based on the (002) peak centers. The thickness of graphitic domains (*L_c_*) is calculated to be in the range of 3.3 nm, indicating that they are consisted of 8 to 9 stacked graphitic layers (i.e. 3.3/0.358 = 9.22). HC-GF inherits this disordered characteristic, which distinguishes it from graphite. The structural anisotropy of electrode materials fundamentally dictates the fast-charging capability of batteries. As illustrated in Fig. S5, in contrast to graphite’s limited edge-to-center pathway, HC enables Li-ion diffusion through open defect sites. This defect-to-center mechanism shortens diffusion paths for superior kinetics, yet HC deployment is hindered by low intrinsic conductivity and loose interparticle contact [32–34].

**Fig. 2.**
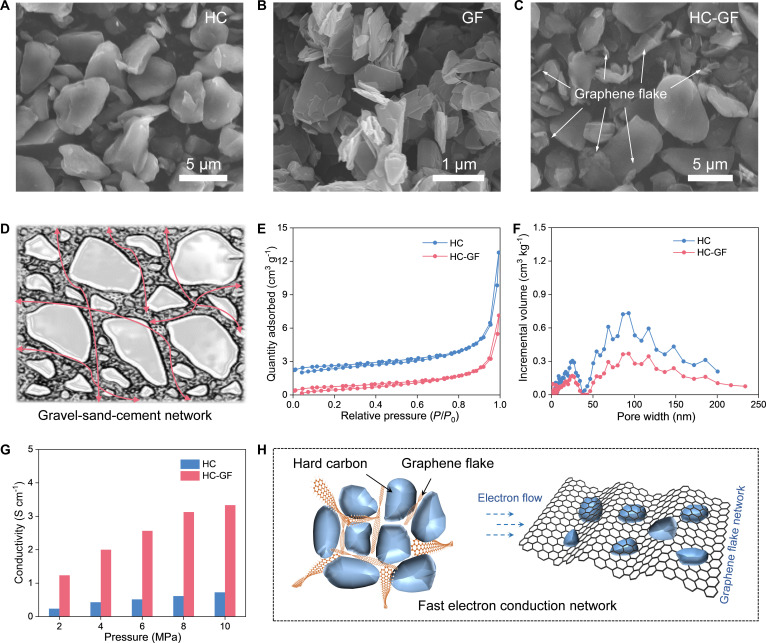
(A to C) SEM images of HC, GF, and HC-GF. (D) Schematic of the “gravel–sand–cement” structure formed by the hard carbon/graphene flake composite. (E) N_2_ adsorption–desorption isotherms and (F) pore size distributions of HC and HC-GF. (G) Electrical conductivity of HC and HC-GF as a function of pressure. (H) Schematic of the fast electron conduction network.

Scanning electron microscopy (SEM) was used to track the morphological evolution of the components as they integrate into a complete system. A “gravel-sand-cement” HC-GF network was constructed as a strategy to improve the conductivity of the HC-based anode. Figure 2A and B and Fig. S6 are SEM images of pure HC and GF material, respectively. The HC phase, acting as the “gravel”, comprises irregular 8- to 10-μm blocks, whereas the GFs feature ultrathin, flexible sheets with high aspect ratios. These distinct morphologies define their complementary roles upon integration via a simple impregnation strategy. As shown in Fig. 2C and D, the existence of GF can be clearly seen in the HC system. The GFs do not merely rest on the surface but conformably wrap around the rigid HC particles and bridge adjacent units. This configuration creates a continuous 3-dimensional (3D) interconnected conductive framework, which mitigates the issue of slow interparticle charge transport under high current densities. Figure 2E and F and Fig. S7 display the nitrogen adsorption–desorption isotherms and pore size distributions. The reduction in specific surface area and porosity for HC-GF material also indicates that the GFs have successfully filled the voids in the accumulation of HC materials [35,36].

A dramatic enhancement in charge transport properties is achieved through morphological and porosity optimization. As displayed in Fig. 2G, which compares the pressure-dependent electrical conductivity of the materials, the HC-GF network exhibits a substantial improvement over pure HC. With the addition of just 5% GFs, the electrical conductivity increases nearly 5-fold. At 10 MPa, it jumps from 0.725 S/cm for pure HC to 3.333 S/cm for the HC-GF composite. As shown in Fig. 2H, unlike the pristine HC, where electrons must navigate a tortuous path through highly resistive point-to-point contacts, the HC-GF network establishes “electron highways”. The GFs bypass these resistance barriers, ensuring rapid electron supply to every active site and significantly lowering the ohmic polarization, thus fully unlocking the intrinsic high-rate potential of the HC anode [35].

**Fig. 3.**
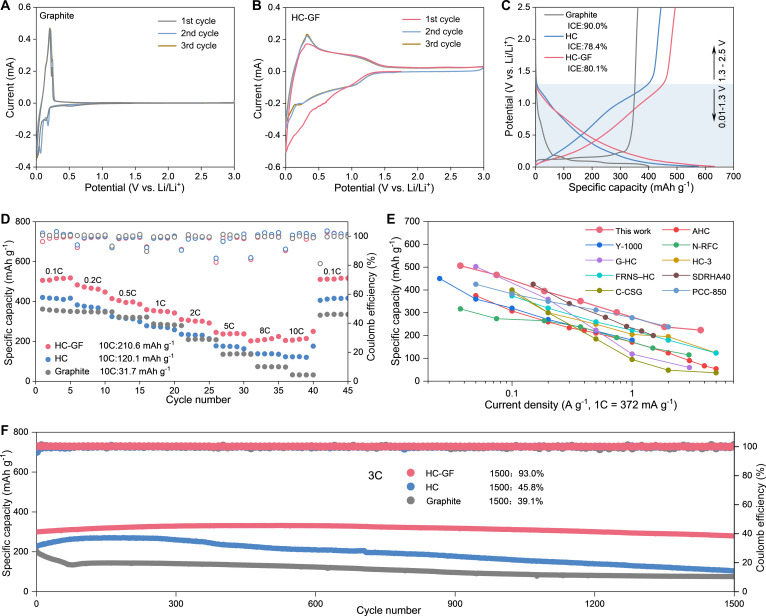
(A and B) Cyclic voltammetry (CV) curves of graphite and HC-GF for the first 3 cycles. (C) Initial charge–discharge profiles of commercial graphite, HC, and HC-GF and (D) comparison of rate performance. (E) Comparison of the initial coulombic efficiency and rate performance between HC-GF and reported Li-ion battery anode materials. (F) Cycling performance of graphite, HC, and HC-GF.

The half-cell tests are carried out by using CR2032 coin-type cells to investigate the electrochemical properties of graphite, HC-GF, and HC materials. Figure 3A and B and Figs. S8 to S10 show the cyclic voltammetry (CV) curves of commercial graphite, HC, and HC-GF electrodes at a scan rate of 0.2 mV s^−1^. A cathodic peak is observed at low potentials during the first cycle, which is likely attributed to the formation of the solid electrolyte interphase (SEI) layer [28,37]. The area enclosed by the CV curve is primarily confined to the voltage range of 0.01 to 1.3 V, reflecting the characteristic behavior of lithium storage in HC materials. Another pair of sharp cathodic (~0.01 V) and anodic peaks (~0.3 V) corresponds to Li-ion intercalation/extraction into carbon interlayers [38,39]. It is noteworthy that the anodic peak of the HC-GF material is more pronounced, indicating a greater prevalence of intercalation mechanisms. Figure 3C and Fig. S11 show the galvanostatic curves of HC, HC-GF, and commercial graphite. The galvanostatic discharge curves of the HC and HC-GF electrodes exhibit a relatively flat discharge plateau, with up to 89.5% of the capacity delivered below 1.3 V. This stable low-voltage plateau is highly favorable for maximizing the operating voltage window in full-cell configurations [40].

Figure 3D displays the rate capability profiles ranging from 0.1 to 10 C. The HC-GF electrode exhibits the highest discharge capacity (506.4 mAh g^−1^) with a high initial coulombic efficiency (ICE) of 80.1%. Furthermore, the HC-GF demonstrates superior fast Li-ion storage capability compared to both the HC and commercial graphite electrodes, particularly at high current densities. The capacity of the graphite electrode is nearly completely attenuated at 10 C (31.7 mAh g^−1^). In contrast, the HC-GF electrode retains capacities of 237.9 and 223.8 mAh g^−1^ at 5 and 10 C, respectively. More notably, the rate performance of HC-GF electrode is much higher than that of previously reported HC anode materials (Fig. 3E) [41–50]. This is of great significance for the practical application of HC-GF materials. Furthermore, the HC-GF electrode exhibits excellent long-term cycling stability. After 1,500 cycles at a rate of 3 C, it maintains a stable reversible capacity of 300.6 mA h g^−1^ with 93.0% capacity retention, demonstrating negligible structural degradation during prolonged electrochemical operation (Fig. 3F).

**Fig. 4.**
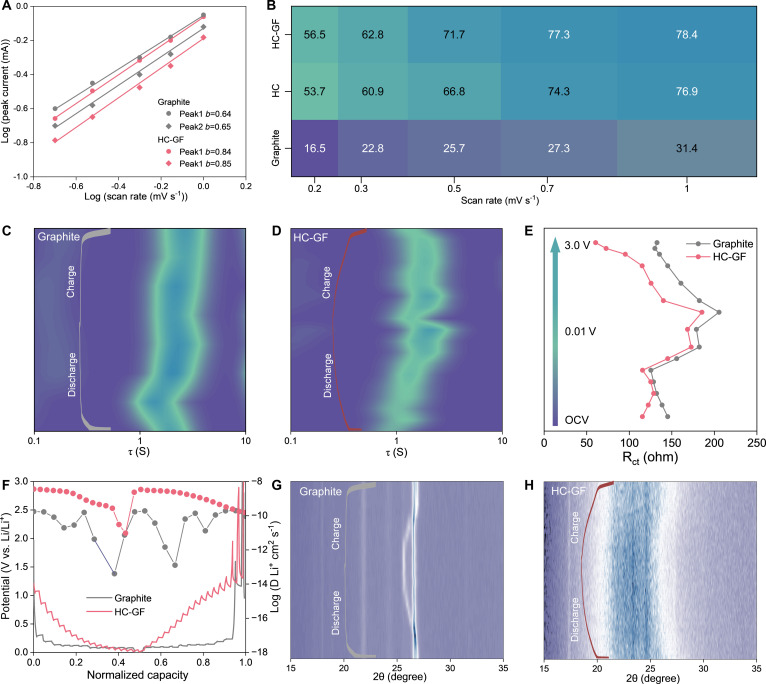
(A) The *b* values and (B) pseudocapacitive contributions based on the CV curves at different scan rates. (C and D) In situ distribution of relaxation times (DRT) analysis of graphite and HC-GF. (E) *R*_ct_ resistance at different voltages. (F) GITT profiles of graphite and HC-GF with the corresponding Li-ion diffusion coefficients. (G and H) In situ XRD patterns of graphite and HC-GF.

The kinetic characteristics of the HC-GF electrode can be evaluated using CV techniques. A series of CV tests were carried out at various scan rates from 0.2 to 1.0 mV s^−1^ (Fig. S12). The relationship between peak current (*i*) and scan rate (*v*) is analyzed using the power–law equation: $i=av^{b}$, where *a* and *b* are adjustable parameters [51,52]. As plotted in Fig. 4A and Fig. S13, the determination of the *b* value provides a metric to distinguish between diffusion-controlled intercalation (*b* ≈ 0.5) and surface-controlled pseudocapacitance (*b* ≈ 1.0). The graphite anode exhibits *b* values of 0.64 (anodic) and 0.65 (cathodic), indicating a kinetics heavily constrained by bulk diffusion. In contrast, the HC-GF anode exhibits higher *b* values of 0.84 and 0.85, which indicates that the charge storage kinetics are governed by surface-induced pseudocapacitance rather than slow bulk diffusion. The capacity controlled contributions can be calculated according to the following equation: $i\left(V\right)=k_{1}v+k_{2}v^{1/2}$, where *k_1_v* and *k_2_v^1/2^* represent pseudocapacitance and diffusion processes, respectively. This kinetic advantage is quantitatively visualized in Fig. 4B. The pseudocapacitive contribution increasingly dominates the total charge storage of the HC-GF anode with increasing scan rate. At a high scan rate of 1.0 mV s^−1^, the capacitive ratio reaches a remarkable 78.4%, which is more than that of the graphite (31.4%) and HC anode (76.9%). This high pseudocapacitive fraction suggests that Li-ions are rapidly stored on the ample surface sites provided by the “electronic Velcro network”, thereby bypassing the sluggish bulk solid-state diffusion pathways [53–55].

The distribution of relaxation times (DRT) analysis, derived from in situ electrochemical impedance spectroscopy (EIS), was employed to visualize the dynamic evolution of resistance during cycling. As shown in the DRT contour maps (Fig. 4C and D), the graphite anode displays a sharp and high-intensity peak in the low-frequency region, while the HC-GF electrode exhibits a smooth and low-intensity feature throughout the entire lithiation/delithiation process. As summarized in Fig. 4E, the total internal resistance of the HC-GF electrode remains consistently lower than that of graphite across the entire voltage window. This validates that the integrated GFs not only enhances bulk conductivity but also mitigates the interfacial charge transfer resistance (*R*_ct_) [56,57].

To further probe the diffusion kinetics, the galvanostatic intermittent titration technique (GITT) is utilized. As shown in Fig. 4F, GITT measurements reveal that the graphite anode suffers from sharp D_Li_^+^ drops to 10^−10^ to 10^−12^ cm^2^ s^−1^ at voltage plateaus, indicating sluggish 2-phase kinetics, whereas HC-GF maintains stable, high values (10^−8^ to 10^−10^ cm^2^ s^−1^) without fluctuations. In situ XRD further corroborated these findings (Fig. 4G and H). Unlike graphite, the HC-GF electrode exhibited negligible shift or intensity variation of its (002) diffraction peak during cycling. This confirms that HC-GF accommodates Li^+^ ions with minimal lattice distortion, ensuring long-term structural integrity even under fast charging conditions.

**Fig. 5.**
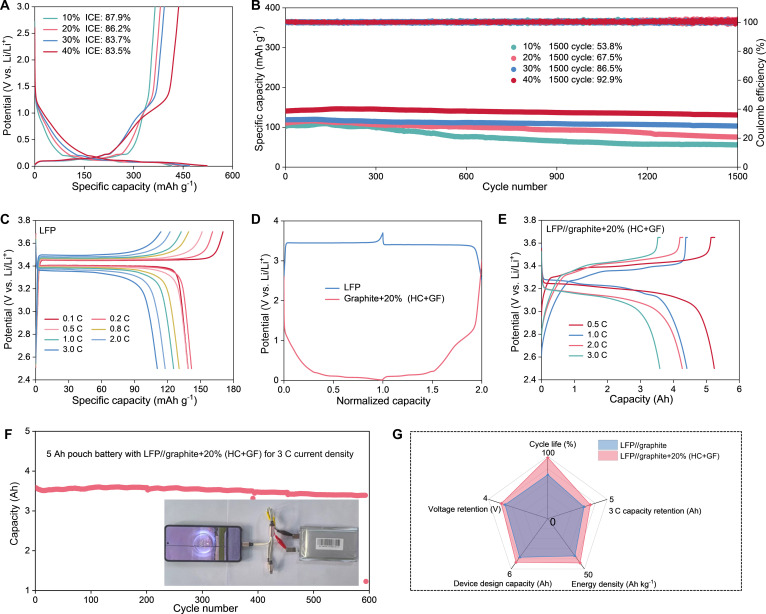
(A) Initial charge–discharge profiles of graphite/HC-GF blended anodes with various mass ratios and (B) the corresponding cycling performance. (C) Charge–discharge profiles of the LiFePO_4_ (LFP) cathode at various rates. (D) Comparison of the normalized voltage profiles between the cathode and anode. (E) Charge–discharge profiles of the 5 Ah LFP//graphite + 20% (HC-GF) pouch battery at various rates. (F) Cycling stability and a digital photograph demonstrating the practical application of powering a smartphone. (G) Comprehensive comparison between LFP//graphite and LFP//graphite + 20% (HC-GF) pouch batteries.

To validate its practical potential, the HC-GF composite was first optimized with graphite in half-cells at varying mass ratios (10% to 40% HC-GF; Fig. 5A). While higher HC-GF content slightly reduced the ICE to 83.5% for the 40% HC-GF blend, it proved essential for enhancing the reversible capacity. Long-term cycling (Fig. 5B) and rate performance (Figs. S14 and S15) also clearly demonstrate this benefit. The graphite + 10% HC-GF anode suffers rapid capacity decay (53.8% retention after 1,500 cycles), while the graphite + 40% HC-GF anode exhibits remarkable durability (92.9% retention). Based on the trade-off among ICE, reversible capacity, and cycling stability, the graphite + 20% HC-GF electrode was selected for pouch battery assembly to explore its practical application.

To validate the optimized formulation, a LFP//graphite + 20% HC-GF pouch battery was assembled using the graphite + 20%HC-GF anode and lithium iron phosphate (LFP) cathode. The charge and discharge curves of the LFP cathode at different rates are shown in Fig. 5C. The capacity ratio between the anode and cathode was precisely matched based on their normalized capacities to maximize energy output (Fig. 5D) [57]. To demonstrate scalability, a 5 Ah LFP//graphite + 20% HC-GF pouch battery was assembled (Figs. S16 to S19). As shown in Fig. 5E and Fig. S20, it delivers exceptional rate capability with stable voltage plateaus with minimal polarization as the current density increases from 0.5 to 3.0 C, retaining a high capacity of 3.58 Ah at 3 C. Furthermore, the pouch battery demonstrates outstanding electrochemical stability under rigorous operational conditions. As depicted in Fig. 5F and Fig. S21, it maintains stable cycling for over 600 cycles at a high current rate of 3.0 C, retaining 94.6% of its initial capacity, which surpasses the performance of commercial LiFePO_4_//graphite pouch battery. Demonstrating its practical viability, the fully charged 5 Ah pouch battery successfully powered a commercial smartphone. Figure 5G presents a radar plot comparing the performance parameters of the 2 pouch batteries. The LFP//graphite + 20% (HC-GF) batteries exhibit superior characteristics in all indicators. These results underscore the introduction of HC-GF as a highly effective strategy for substantially enhancing the overall performance of LIBs.

## Conclusion

This work bridges laboratory synthesis and industrial application by presenting a kilogram-scale HC-GF composite anode designed for ultra-fast charging LIBs. Through the “electronic Velcro” network design concept, highly conductive GFs are intimately integrated with the HC matrix (HC-GF). This HC-GF composite not only adapts to the structural evolution but also significantly enhances the electronic conductivity of the electrode material. Consequently, the HC-GF composite delivers exceptional capacity at extreme current densities (223.8 mAh g^−1^ at 10 C). Critically, practical viability is demonstrated in a 5 Ah-level pouch cell with graphite-blended anode, achieving stable 3 C cycling and successfully powering commercial electronics. This work provides a scalable and scientifically robust pathway for next-generation high-power energy storage systems.

## Methods

### Material synthesis

Petroleum coke precursor was mechanically pulverized and sieved to obtain powder with a particle size of approximately 10 μm. The powder was then placed in an atmosphere box furnace and heated to 900 °C for 1.5 h at a rate of 5 °C min^−1^ under a nitrogen atmosphere. After naturally cooling to room temperature, the resulting product was denoted as HC. Subsequently, the HC-GF composite was synthesized via a facile impregnation strategy to form an integrated conductive network.

### Material characterization

The surface morphology and internal microstructure of the as-prepared samples were characterized using SEM (Hitachi S-4800 operated at 10 kV) and HRTEM (Tecnai G2 F20 operated at 200 kV). To quantitatively analyze the microstructural evolution, ArcGIS software was utilized to process HRTEM images. Crystallographic structures and phase purity were investigated using XRD (Bruker D8 Advance with Cu-Kα radiation). Raman spectroscopy (Lab RAM HR800 with an excitation laser wavelength of 532 nm, an effective laser power on the sample of 5 mW) was employed to assess the degree of graphitization and the density of structural defects. The specific surface area and pore size distribution were determined by nitrogen adsorption–desorption isotherms using the Brunauer–Emmett–Teller method (a Micromeritics 3 Flex surface characterization analyzer at 77 K using nitrogen as the adsorbate). To evaluate electrical properties under realistic electrode conditions, the powder conductivity of the materials was measured under varying compaction pressures (ranging from 0 to 10 MPa). The measurements were repeated 3 times with an error smaller than 3%. Furthermore, in situ XRD mapping was conducted during electrochemical cycling to monitor real-time structural evolution and lattice strain during the lithiation and delithiation processes.

### Electrochemical measurements

Electrochemical properties were evaluated in CR2032 coin-type half-cells. The slurry was prepared by mixing active material (HC, graphite, or HC-GF composites), acetylene black, and poly(vinylidene fluoride) with mass ratio of 9.0:0.5:0.5 in N-methyl-2-pyrrolidinone. The working electrode was assembled with a lithium metal foil serving as both the counter and reference electrode. Electrolyte was 1 M LiPF_6_ dissolved in ethylene carbonate and diethyl carbonate (EC:DEC = 1:1 in volume ratio). CV measurements were performed within a voltage window of 0.01 to 3.0 V (versus Li/Li^+^) at scan rates ranging from 0.2 to 1.0 mV s^−1^. Rate capability was assessed at current densities from 0.1 to 10 C. Long-term cycling stability was tested at a high rate of 3 C for 1,500 cycles. EIS was conducted with an alternating current (AC) signal of 10 mV (rms) with 0.1 to 100,000 Hz varying frequencies to analyze interfacial charge transfer resistance. The obtained spectra were further processed using DRT. GITT was employed to quantify the lithium-ion diffusion coefficients based on the following equation: $D=\frac{4}{\pi \tau }{\left(\frac{m_{B}V_{m}}{M_{B}S}\right)}^{2}\left(\frac{∆E_{s}}{∆E_{\tau }}\right)$, where *m_B_* is the mass of the electrode material, *V_m_* and *M_B_* are the molar volume and molar mass of the electrode materials, *S* is the geometric area of the electrode, τ is the period of the pulse, *ΔE_τ_* is the total transient voltage change of the half-cell for an applied galvanostatic pulsed current *I* for the time *τ*, and *ΔE_s_* is the change of the steady-state voltage of the half-cell for this step. To validate the industrial scalability and practical feasibility of the proposed electrode strategy, 5 Ah-level soft-pack pouch batteries were fabricated using the optimized blended material (commercial graphite mixed with 20 wt % HC-GF) as the anode and commercial lithium iron phosphate (LiFePO_4_) as the cathode. During the assembly process, the negative-to-positive (N/P) capacity ratio was meticulously adjusted to ensure that the anode operated within a safe potential window, effectively preventing lithium plating. The electrochemical performance of these full cells was rigorously evaluated, including rate capability tests ranging from 0.5 to 2.0 C and cycling stability assessments conducted at a high rate of 3.0 C.

## Data Availability

All data are available in the manuscript or the Supplementary Materials or from the authors.

## References

[1] Li H, Bin Kaleem M, Liu KL, Wu Y, Liu WR, Peng Q. Fault prognosis of Li-ion batteries in electric vehicles: Recent progress, challenges and prospects. J Energy Storage. 2025;116: Article 116002.

[2] Geslin A, Xu L, Ganapathi D, Moy K, Chueh WC, Onori S. Dynamic cycling enhances battery lifetime. Nat Energy. 2025;10(2):172–180.

[3] Min XQ, Xu GJ, Xie B, Guan P, Sun ML, Cui GL. Challenges of prelithiation strategies for next generation high energy lithium-ion batteries. Energy Storage Mater. 2022;47:297–318.

[4] Zhou JW, Li Y, Dong YT, Shen HY, Xu XJ, Yue XY, Tao XY, Liang Z, Zhang D. Curvature-induced reversible Li plating behavior enables extremely fast-charging and long-lasting Li-ion batteries. ACS Nano. 2025;19(47):40506–40515. 41251135 10.1021/acsnano.5c14714

[5] Fan TE, Gao P, Wan Y, Qu BH. Optimized fast-charging of lithium-ion battery packs via predictive-reinforcement hierarchical control framework. J Energy Storage. 2026;151: Article 120395.

[6] Xiao H, Zhao JT, Gao QX, Zhang WJ, Cheng X, Song CY, Li GX. Recent advances in fast-charging lithium-ion batteries: Mechanism, materials, and future opportunities. Chem Eng J. 2025;506: Article 159927.

[7] Lefa I, Farmakis F. Front-runner anode materials in the fast-charging race of lithium-ion batteries: A review of recent advances. J Power Sources. 2026;662.

[8] Qiao XJ, Wang LG, Lu J. The tuning of strain in layered structure oxide cathodes for lithium-ion batteries. Research. 2024;7: Article 0489. 39296985 10.34133/research.0489 PMC11409455

[9] Wong WL, Xu JH, Zhao Y, Wang YD, Du H, Zhang JH, Kang YQ, Chen YQ, Kang FY, Li BH. Upcycling of degraded Prussian blue into layered materials for sodium-ion battery. Research. 2025;8: Article 0643. 40123995 10.34133/research.0643 PMC11927955

[10] Jia K, Yang GR, He YJ, Cao ZJ, Gao JT, Zhao HY, Piao ZH, Wang JX, Abdelkader AM, Liang Z, et al. Degradation mechanisms of electrodes promotes direct regeneration of spent Li-ion batteries: A review. Adv Mater. 2024;36(23): Article 2133273. 10.1002/adma.202313273 38533901

[11] Jiang XM, Chen YJ, Meng XK, Cao WG, Liu CC, Huang Q, Naik N, Murugadoss V, Huang MA, Guo ZH. The impact of electrode with carbon materials on safety performance of lithium-ion batteries: A review. Carbon. 2022;191:448–470.

[12] Zhao LF, Hu Z, Lai WH, Tao Y, Peng J, Miao ZC, Wang YX, Chou SL, Liu HK, Dou SX. Hard carbon anodes: Fundamental understanding and commercial perspectives for Na-ion batteries beyond Li-ion and K-ion counterparts. Adv Energy Mater. 2021;11(1): Article 2002704.

[13] Tang Z, Zhou SY, Huang YC, Wang H, Zhang R, Wang Q, Sun D, Tang YG, Wang HY. Improving the initial coulombic efficiency of carbonaceous materials for Li/Na-ion batteries: Origins, solutions, and perspectives. Electrochem Energy Rev. 2023;6(1):8.

[14] Lege N, He XX, Wang YX, Lei YJ, Yang YX, Xu JT, Liu M, Wu XQ, Lai WH, Chou SL. Reappraisal of hard carbon anodes for practical lithium/sodium-ion batteries from the perspective of full-cell matters. Energy Environ Sci. 2023;16(12):5688–5720.

[15] Li YQ, Vasileiadis A, Zhou Q, Lu YX, Meng QS, Li Y, Ombrini P, Zhao JB, Chen Z, Niu YS, et al. Origin of fast charging in hard carbon anodes. Nat Energy. 2024;9(2):134–142.

[16] Chng CJ, Ma XY, Abe Y, Kumagai S. Hard carbon/graphite composite anode for durable lithium-ion capacitor. J Energy Storage. 2024;92: Article 112193.

[17] Alvin S, Cahyadi HS, Hwang J, Chang W, Kwak SK, Kim J. Revealing the intercalation mechanisms of lithium, sodium, and potassium in hard carbon. Adv Energy Mater. 2020;10(20): Article 2000283.

[18] Xie LJ, Tang C, Bi ZH, Song MX, Fan YF, Yan C, Li XM, Su FY, Zhang Q, Chen CM. Hard carbon anodes for next-generation Li-ion batteries review and perspective. Adv Energy Mater. 2021;11(38): Article 2101650.

[19] Xie YZ, Wang YQ, Yamauchi Y, Kim M, Yuan F, He YH, Wu YQ, Wan CC. Synergistic ultramicropore-confined and electronic-state modulation strategies in sustainable lignin-derived hard carbon for robust sodium-ion batteries. Research. 2026;9: Article 1039. 41551911 10.34133/research.1039 PMC12804597

[20] Li Q, Liu XS, Tao Y, Huang JX, Zhang J, Yang CP, Zhang YB, Zhang SW, Jia YR, Lin QW, et al. Sieving carbons promise practical anodes with extensible low-potential plateaus for sodium batteries. Natl Sci Rev. 2022;9(8): Article nwac084. 35992230 10.1093/nsr/nwac084 PMC9385462

[21] Liu YY, Shi HD, Wu ZS. Recent status, key strategies and challenging perspectives of fast-charging graphite anodes for lithium-ion batteries. Energy Environ Sci. 2023;16(11):4834–4871.

[22] Chen C, Huang Y, Lu MW, Zhang JX, Li TH. Tuning morphology, defects and functional group types in hard carbon via phosphorus doped for rapid sodium storage. Carbon. 2021;183:415–427.

[23] Liu L, Du ML, Li G, Schobert HH, Fan JW, Liu J, Wang Q. Structure and evolution features of cutinite with different coal rank from stacking and arrangement of aromatic fringes in HRTEM. Fuel. 2022;326: Article 124998.

[24] Wang XL, Wang SQ, Hao C, Zhao YG, Song XX. Quantifying orientation and curvature in HRTEM lattice fringe micrographs of naturally thermally altered coals: New insights from a structural evolution perspective. Fuel. 2022;309: Article 122180.

[25] Lv T, Zhang J, Yang XM, Qiu JS. Soft-hard heterostructure functional carbon materials: Synthesis, structure regulation, and applications in energy storage. Carbon. 2026;247: Article 120958.

[26] Okabe J, Fang Y, Moriguchi I, Furó I. Structural evolution by heat treatment of soft and hard carbons as Li storage materials: A joint NMR/XRD/TEM/Raman study. J Mater Chem A. 2025;13(19):13962–13975.

[27] Tao RM, Steinhoff B, Sun XG, Sardo K, Skelly B, Meyer HM III, Sawicki C, Polizos G, Lyu X, Du ZJ, et al. High-throughput and high-performance lithium-ion batteries via dry processing. Chem Eng J. 2023;471: Article 14430.

[28] Cui YP, Liu W, Feng WT, Zhang Y, Du YX, Liu S, Wang HL, Chen M, Zhou JA. Controlled design of well-dispersed ultrathin MoS2 nanosheets inside hollow carbon skeleton: Toward fast potassium storage by constructing spacious “houses” for K ions. Adv Funct Mater. 2020;30(10): Article 1908755.

[29] Peng H, Long TR, Peng J, Chen H, Ji LF, Sun H, Huang L, Sun SG. Molecular design for in-situ polymerized solid polymer electrolytes enabling stable cycling of lithium metal batteries. Adv Energy Mater. 2024;14(22): Article 2400428.

[30] Zhao WB, Zhao CH, Wu H, Li LJ, Zhang CC. Progress, challenge and perspective of graphite-based anode materials for lithium batteries: A review. J Energy Storage. 2024;81: Article 110409.

[31] Wei ZQ, Kong DW, Quan LJ, He JR, Liu JY, Tang ZY, Chen S, Cai QQ, Zhang RQ, Liu HJ, et al. Removing electrochemical constraints on polytetrafluoroethylene as dry-process binder for high-loading graphite anodes. Joule. 2024;8(5):1350–1363.

[32] Liu BY, Li MZ, Wu YJ, Ren R, Ge QL, Fan LZ, Qin P, Qian J, Ding XL. High-entropy BiSnSbCuAl nanoalloys conformed in carbon fibers as fast-charging and high-capacity anode material for sodium-ion batteries. J Power Sources. 2025;652: Article 237600.

[33] Song YJ, Cui YP, Geng L, Li BY, Ge LN, Zhou L, Qiu ZJ, Nan J, Wu W, Xu H, et al. Li/Ni intermixing: The real origin of lattice oxygen stability in Co-free Ni-rich cathode materials. Adv Energy Mater. 2024;14(7): Article 2303207.

[34] Feng WT, Wei XR, Cao FL, Li YT, Zhang XH, Li YP, Liu W, Han JW, Kong DB, Zhi LJ. Defective MoSSe with local-expanded structure for high-rate potassium ion battery. Energy Storage Mater. 2024;65: Article 103186.

[35] Kang S, Ma G, Liu YX, Wang DM, Zhang Y, Li JJ, Xu C, Li YF. Progress in fast-charging graphite anodes for lithium-ion batteries: Reaction kinetics. Energy Storage Mater. 2025;81: Article 104531.

[36] Zhou W, Mo Y, Gao P, Wang KX, Ke JL, Liu Z, Chen S, Liu JL. Decoupling interfacial kinetics realizes 5C fast charging of potassium-ion batteries using graphite anode. Adv Funct Mater. 2024;34(21): Article 2312994.

[37] Cui Y, Liu W, Wang X, Li J, Zhang Y, Du Y, Liu S, Wang H, Feng W, Chen M. Bioinspired mineralization under freezing conditions: An approach to fabricate porous carbons with complicated architecture and superior K^+^ storage performance. ACS Nano. 2019;13(10):11582–11592. 31560191 10.1021/acsnano.9b05284

[38] Liu AL, Li WK, Han B, Ridley P, Ah L, Bhamwala B, Vicencio M, Kannan DRR, Rikka VR, Premnath V, et al. Unveiling the graphite electrolyte interphase evolution under fast charging conditions in commercial cells. ACS Appl Mater Interfaces. 2025;17(50):68650–68660. 41329922 10.1021/acsami.5c17267 PMC12723637

[39] Ma D, Kim DH, Yu M, Cho YE, Kim H. Porous silicon layer overlaid graphite anode materials for fast-charging of lithium-ion batteries. J Power Sources. 2025;645: Article 237199.

[40] Ge L, Song Y, Niu P, Li B, Zhou L, Feng W, Ma C, Li X, Kong D, Yan Z, et al. Elaborating the crystal water of Prussian blue for outstanding performance of sodium ion batteries. ACS Nano. 2024;18(4):3542–3552. 38215406 10.1021/acsnano.3c11169

[41] Yu M, Wang M, Indris S, Manassa J, Stangel A, Hovden R, Laine RM. An unexpected source of hard carbon, rice hull ash, provides unexpected Li+ storage capacities. Adv Sustainable Syst. 2024;9(2): Article 2400667.

[42] Liu S, Zhang K, Lei L, Yin D, Teng J, Wang Q, Tang X, Gao Y, Cai X, Li J. Two-step optimization of hard carbon anodes via carbonisation temperature and chemical vapor deposition for enhanced initial coulombic efficiency and rate performance in Li-ion batteries. Carbon. 2026;247: Article 121022.

[43] Wang P, Gao X, Liang J, Yang S, Liu S, Gong X. Defect vacancies in pitch-based hard carbon coating for a long-life nature graphite anode. Energy Fuel. 2025;39(17):8308–8318.

[44] Xiao Z, Zheng F, Wang Z, Sun Y, Li Z, Sun D, Liao S, Pan T, Ye Q, Xu C, et al. Regulating hard carbon microstructures on graphite anode toward splendid lithium-ion storage performance below 0.5 V. Energy Storage Mater. 2026;84: Article 104777.

[45] Guo X, Qiao Y, Yi Z, Pedersen CM, Wang Y, Tian X, Han P. Furfural residues derived nitrogen-sulfur co-doped sheet-like carbon: An excellent electrode for dual carbon lithium-ion capacitors. Green Energy Environ. 2024;9(9):1427–1439.

[46] Naik PB, Reddy NS, Nataraj SK, Ghosh D. Synergistic stabilization of long-cycling Li-ion battery anodes using biomass-derived silica-embedded hard carbon. J Energy Storage. 2025;126: Article 117067.

[47] Gaikwad M, Pappu S, Pathak A, Sharma C, Kumta PN. Enhanced electrochemical performance and extended cycling of resorcinol-formaldehyde derived N-doped carbon xerogel for alkali metal-ion (Li/Na/K) batteries. J Power Sources. 2026;667: Article 239254.

[48] Qiu Z, Cui Y, Wang D, Wang Y, Hu H, Li X, Cai T, Gao X, Hu H, Wu M, et al. Dual carbon Li-ion capacitor with high energy density and ultralong cycling life at a wide voltage window. Sci China Mater. 2022;65:2373–2384.

[49] Feng X, Wu F, Fu Y, Li Y, Gong Y, Ma X, Zhang P, Wu C, Bai Y. Revealing the effect of curvature structure in hard carbon anodes for lithium/sodium ion batteries. Small. 2025;21(2): Article e2409120. 39558691 10.1002/smll.202409120

[50] Hsu W-C, Babu SK, Hsieh C-T, Liu W-R. Enhanced electrochemical properties of hard carbon anode derived from phenolic resin modified via an oxygen-induced plasma surface treatment for lithium-ion batteries. Surf Interfaces. 2025;62: Article 106282.

[51] Rahman MM, Dixit M, Amin R, Abouimrane A, Kweon CM, Belharouak I. Distinct dynamics of lithium intercalation and plating on graphite anode for Li-ion batteries in eVTOL applications. Adv Energy Mater. 2025;15(40): Article e02538.

[52] Cui Y, Feng W, Wang D, Wang Y, Liu W, Wang H, Jin Y, Yan Y, Hu H, Wu M, et al. Water-soluble salt template-assisted anchor of hollow FeS2 nanoparticle inside 3D carbon skeleton to achieve fast potassium-ion storage. Adv Energy Mater. 2021;11(33): Article 2101343.

[53] Xu YJ, Song XH, Chang Q, Longhou X, Sun Y, Feng XY, Wang XR, Zhan M, Xiang HF, Yu Y. The regeneration of graphite anode from spent lithium-ion batteries via interface washing. New Carbon Mater. 2022;37(5):1011–1020.

[54] Feng WT, Chang B, Ren YF, Kong DB, Tao HB, Zhi LJ, Khan MA, Aleisa R, Rueping M, Zhang HB. Proton exchange membrane water splitting: Advances in electrode structure and mass-charge transport optimization. Adv Mater. 2025;37(15): Article 246012. 10.1002/adma.202416012 PMC12004895 40035170

[55] Feng WT, Wei XR, Yang JH, Ma CY, Sun YM, Han JW, Kong DB, Zhi LJ. Iodine-induced self-depassivation strategy to improve reversible kinetics in Na-Cl2 battery. Nat Commun. 2024;15(1): Article 2416012. 10.1038/s41467-024-51033-1 PMC11319802 39134537

[56] He JH, Meng JK, Huang YH. Challenges and recent progress in fast-charging lithium-ion battery materials. J Power Sources. 2023;570: Article 232965.

[57] Zhao L, Ding BC, Qin XY, Wang ZJ, Lv W, He YB, Yang QH, Kang FY. Revisiting the roles of natural graphite in ongoing lithium-ion batteries. Adv Mater. 2022;34(18): Article 2106704. 10.1002/adma.202106704 35032965

