# Supplementary material for: Functionalization of Fast-Charging Hard Carbon Anode for Ah-Level Li-Ion Pouch Batteries
Source: Research (Wash D C). 2026 Apr 7;9:1238. doi: 10.34133/research.1238 (PMC13053927; doi:10.34133/research.1238)
Supplement: Supplementary 1 — Figs. S1 to S21 [file research.1238.f1.docx]

**Functionalization of fast-charging hard carbon anode for Ah-level Li-ion pouch batteries**


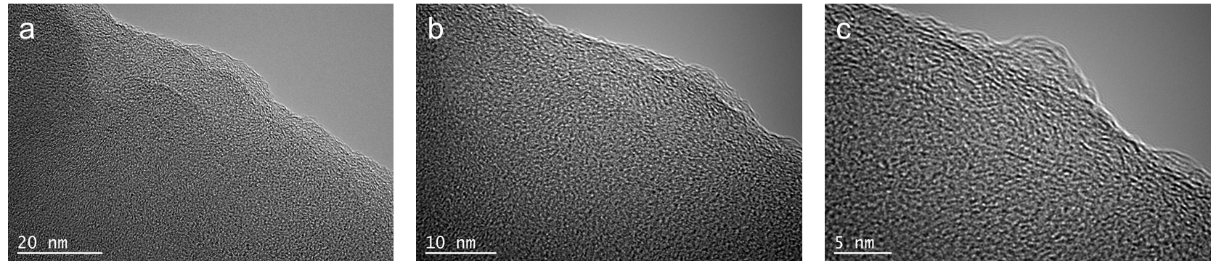


Figure S1. HRTEM images of HC at different magnifications.


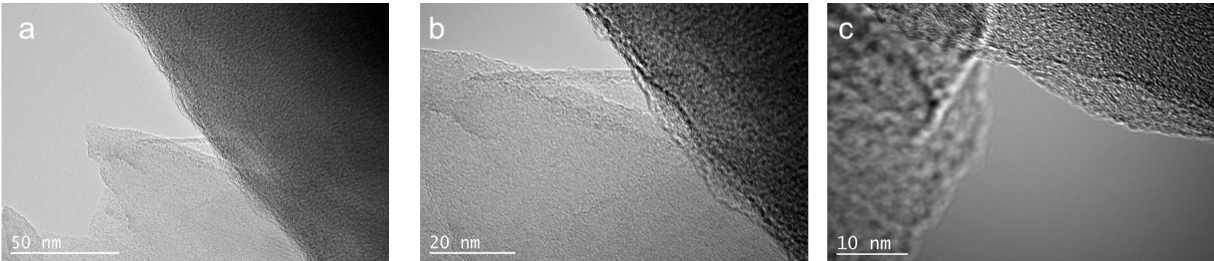


Figure S2. HRTEM images of HC-GF at different magnifications.


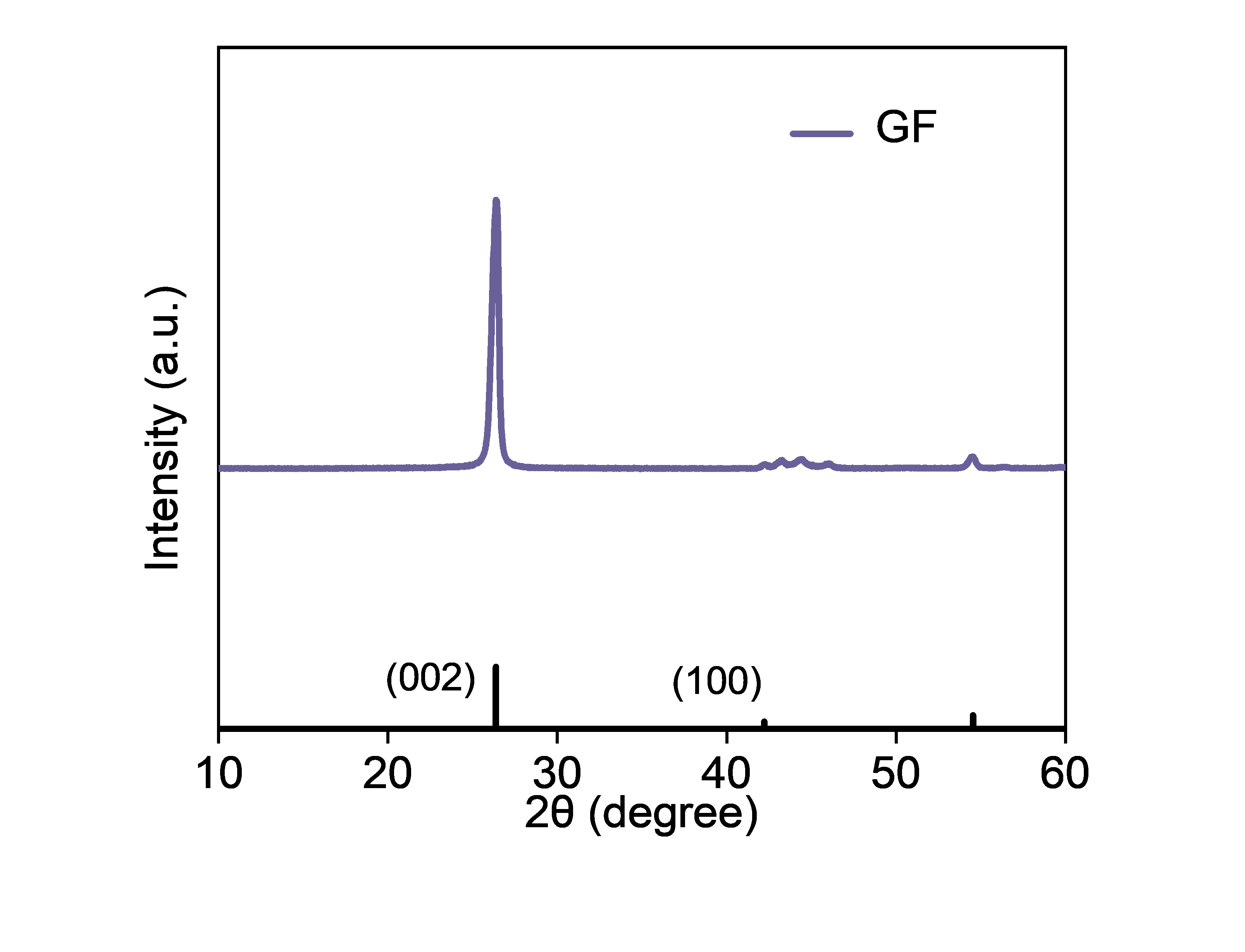


Figure S3. XRD patterns of GF.


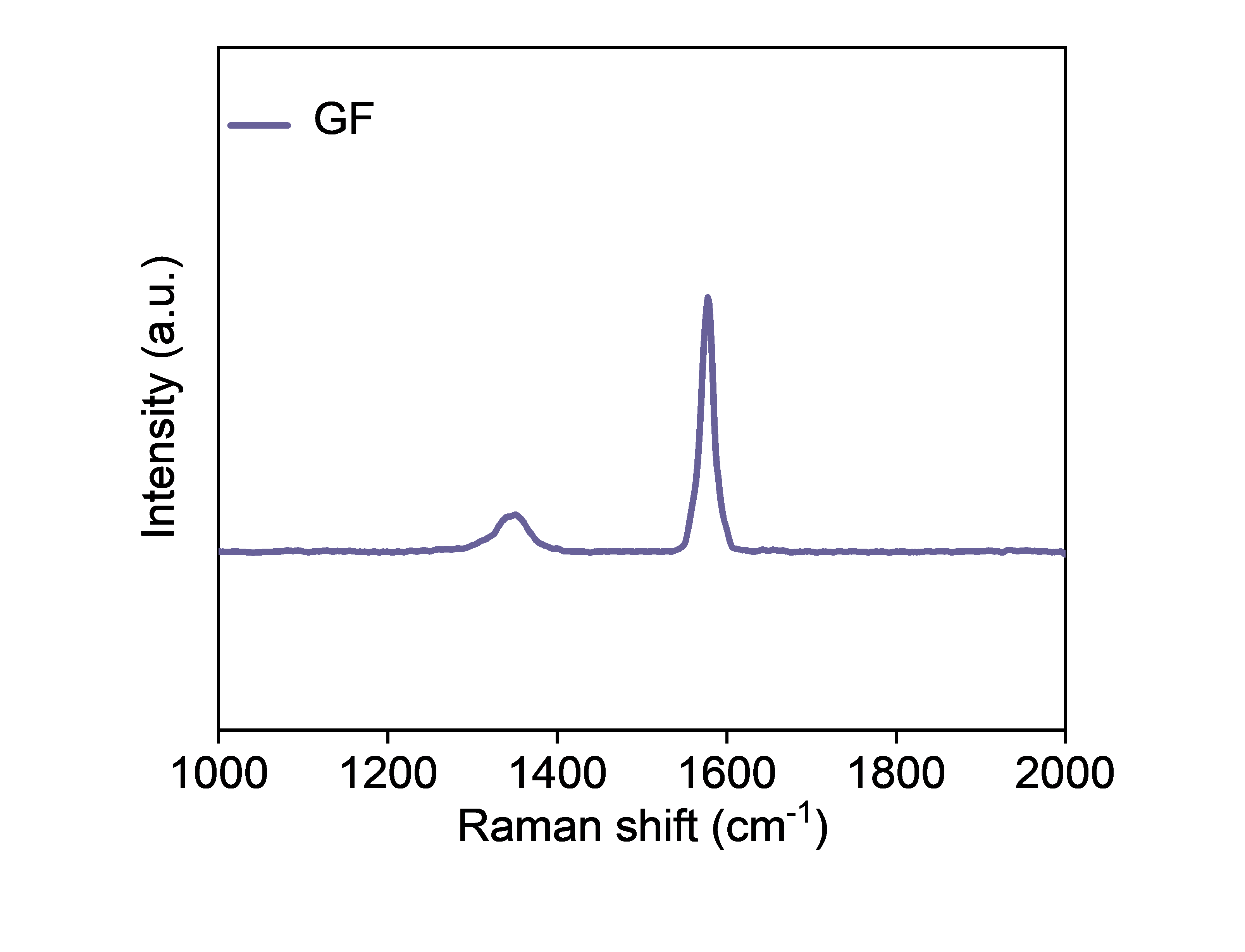


Figure S4. Raman spectra of GF.


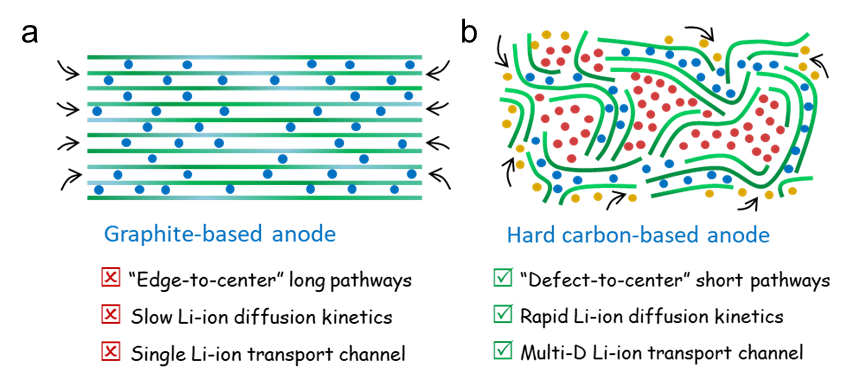


Figure S5. Schematic illustrations of Li-ion transport in graphite and hard carbon anodes.

Note: The structural differences of electrode materials determine the fast-charging performance of battery. As shown in Figure S5a and S5b, for the graphite-based anode, Li-ions can only be intercalated into the ordered layered structure through edge sides. In contrast, hard carbon anode enable Li-ions to cross the layered plane through the open angular defect sites rather than the edge sides, thus avoiding the long-distance “edge-to-center” diffusion path of Li-ions. This multi-dimensional transport channel and “defect-to-center” short-distance diffusion path greatly accelerates the diffusion kinetics of Li-ions in the electrode material, thereby achieving fast charging performance.


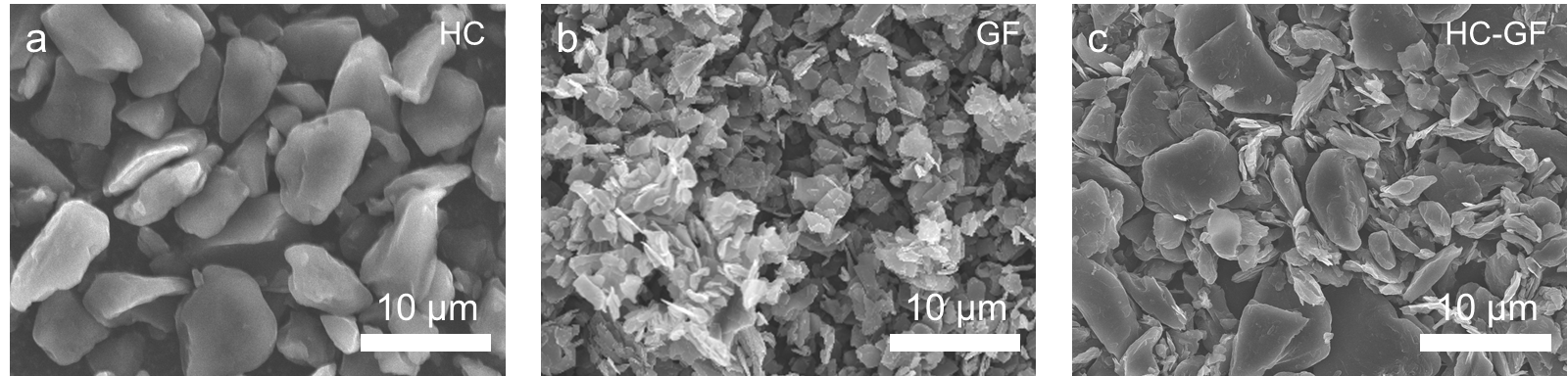


Figure S6. SEM images of hard carbon (HC), graphene flake (GF), and HC-GF.


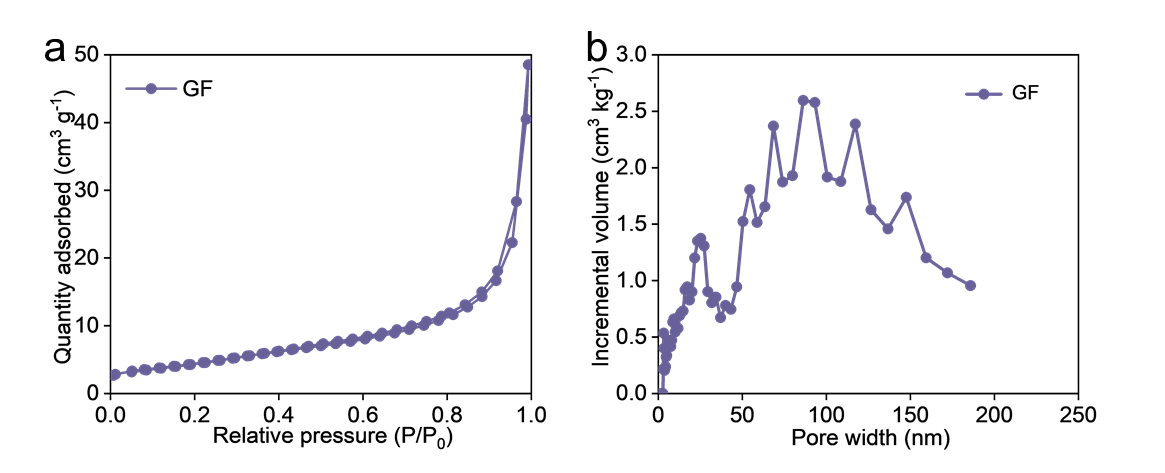


Figure S7. (a) N_2_ adsorption-desorption isotherms and (b) pore size distributions of GF.


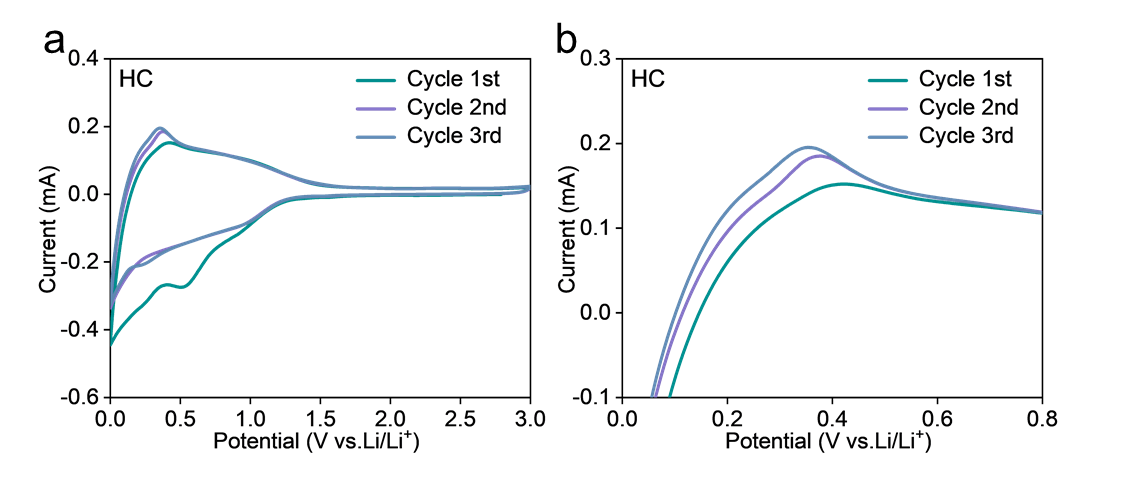


Figure S8. Cyclic voltammetry (CV) curves of HC for the first three cycles.


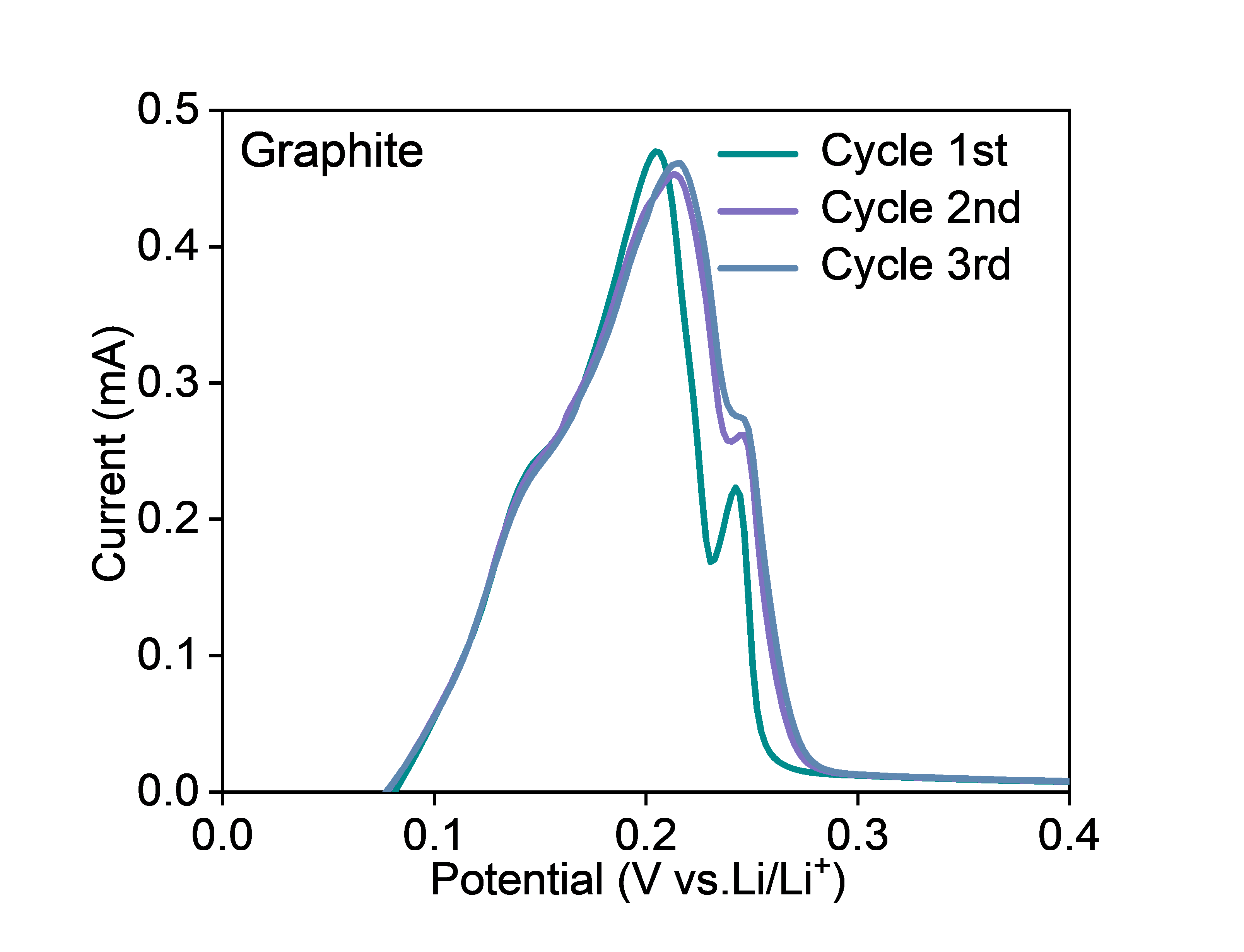


Figure S9. Cyclic voltammetry (CV) curves of graphite for the first three cycles.


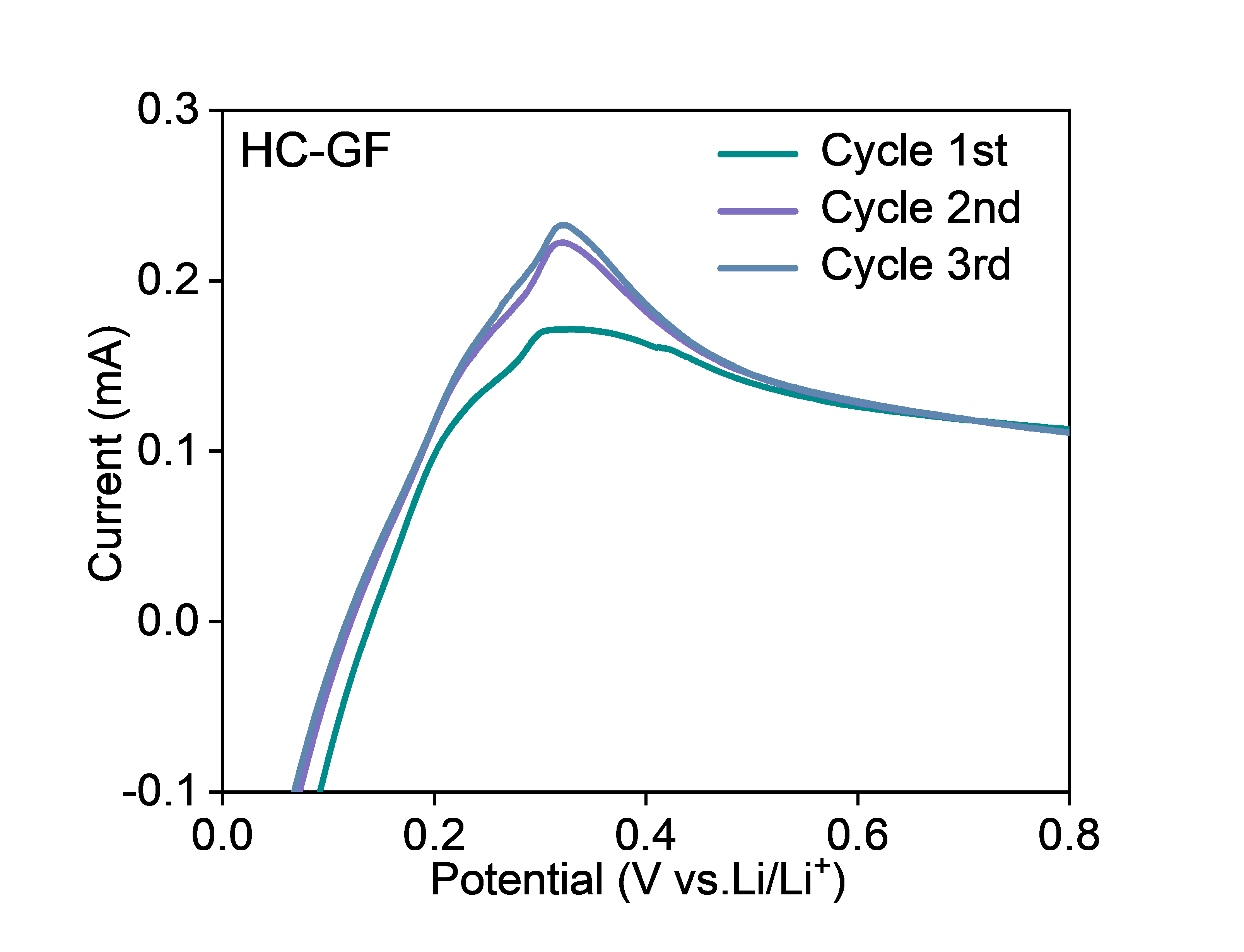


Figure S10. Cyclic voltammetry (CV) curves of HC-GF for the first three cycles.


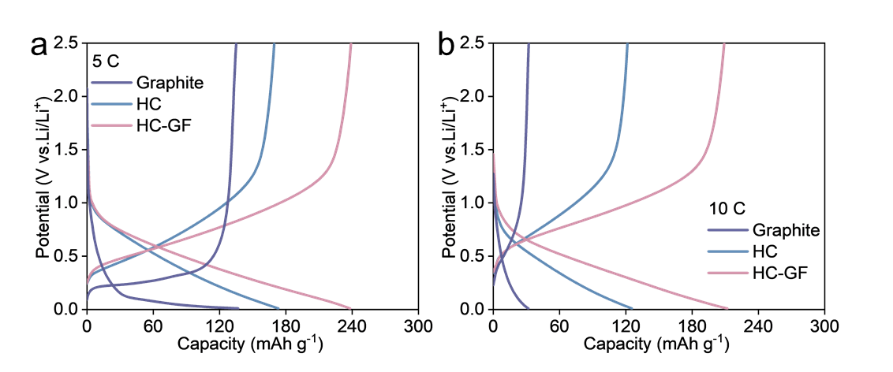


Figure S11. Charge-discharge profiles of HC-GF, graphite, and HC at (a) 5C and (b) 10C.


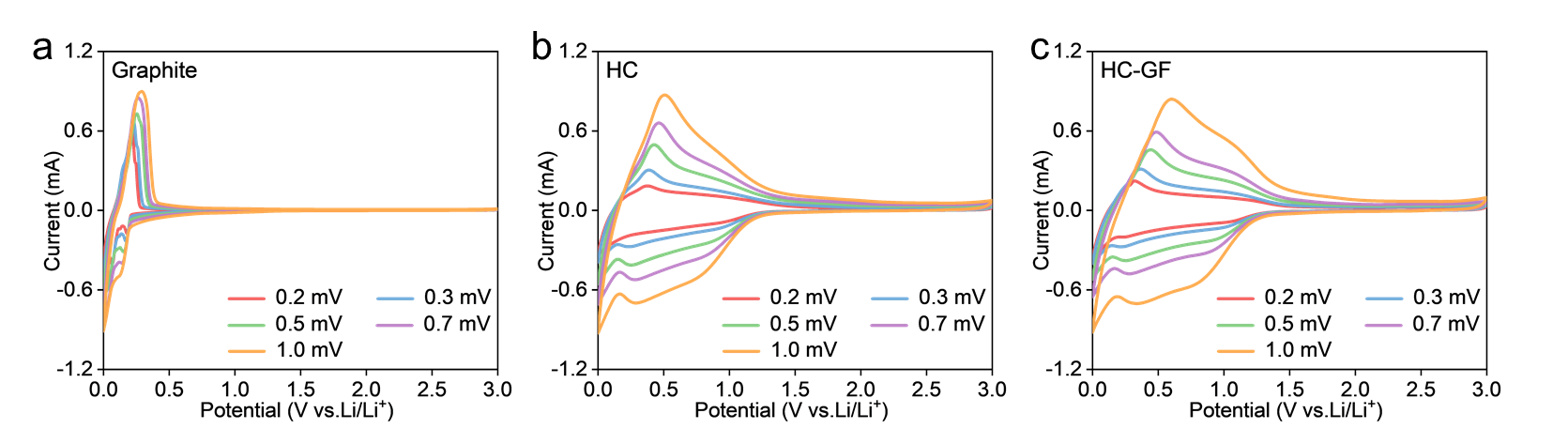


Figure S12. CV curves of graphite, HC and HC-GF at various scan rates.


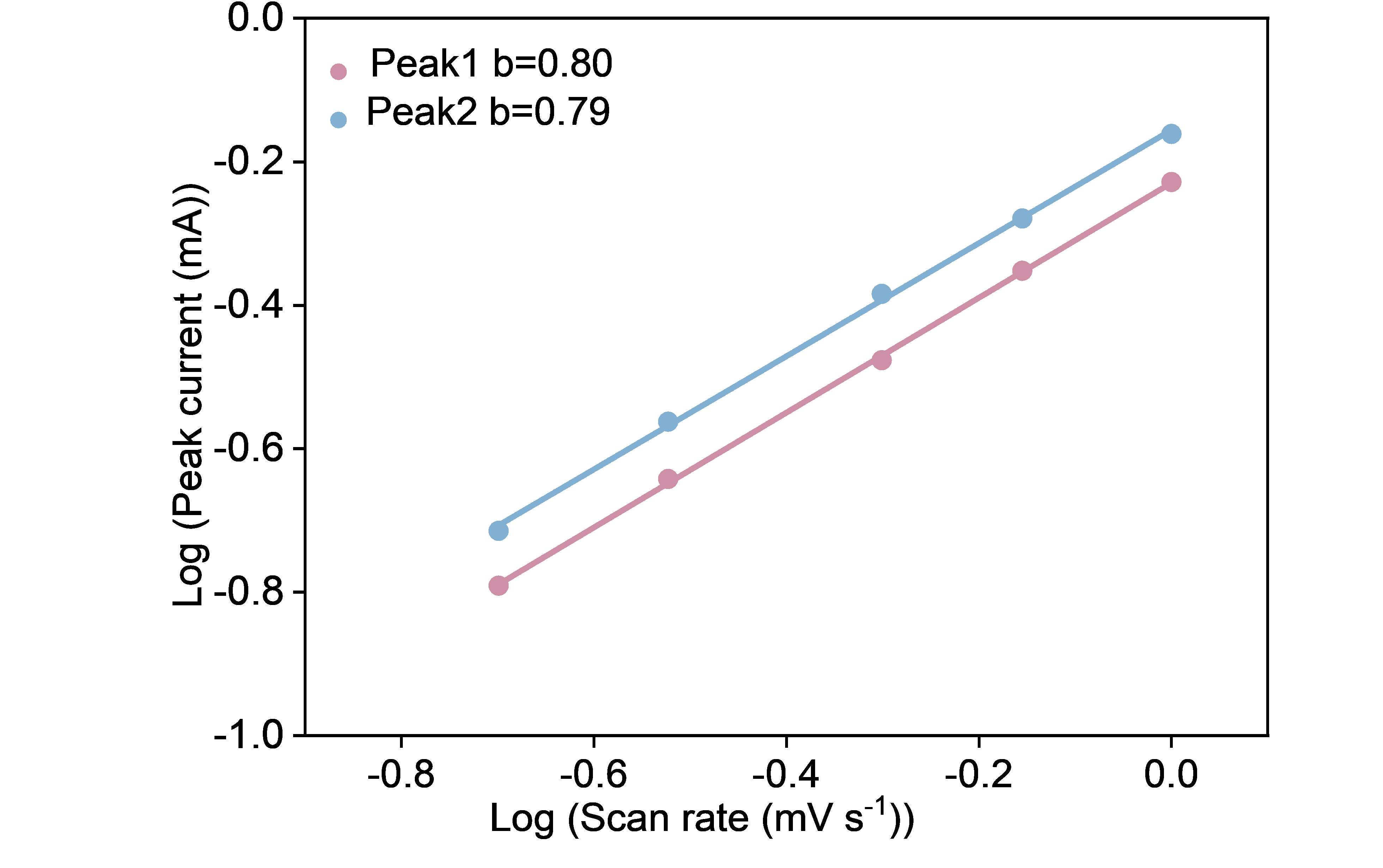


Figure S13. Calculated b-values of HC.


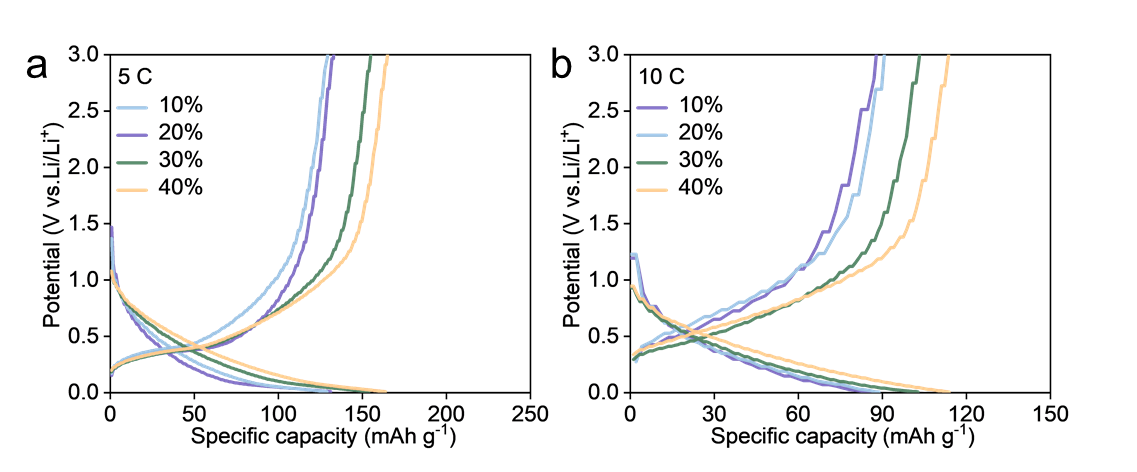


Figure S14. Charge-discharge profiles of graphite/HC-GF blended anodes with various mass ratios.


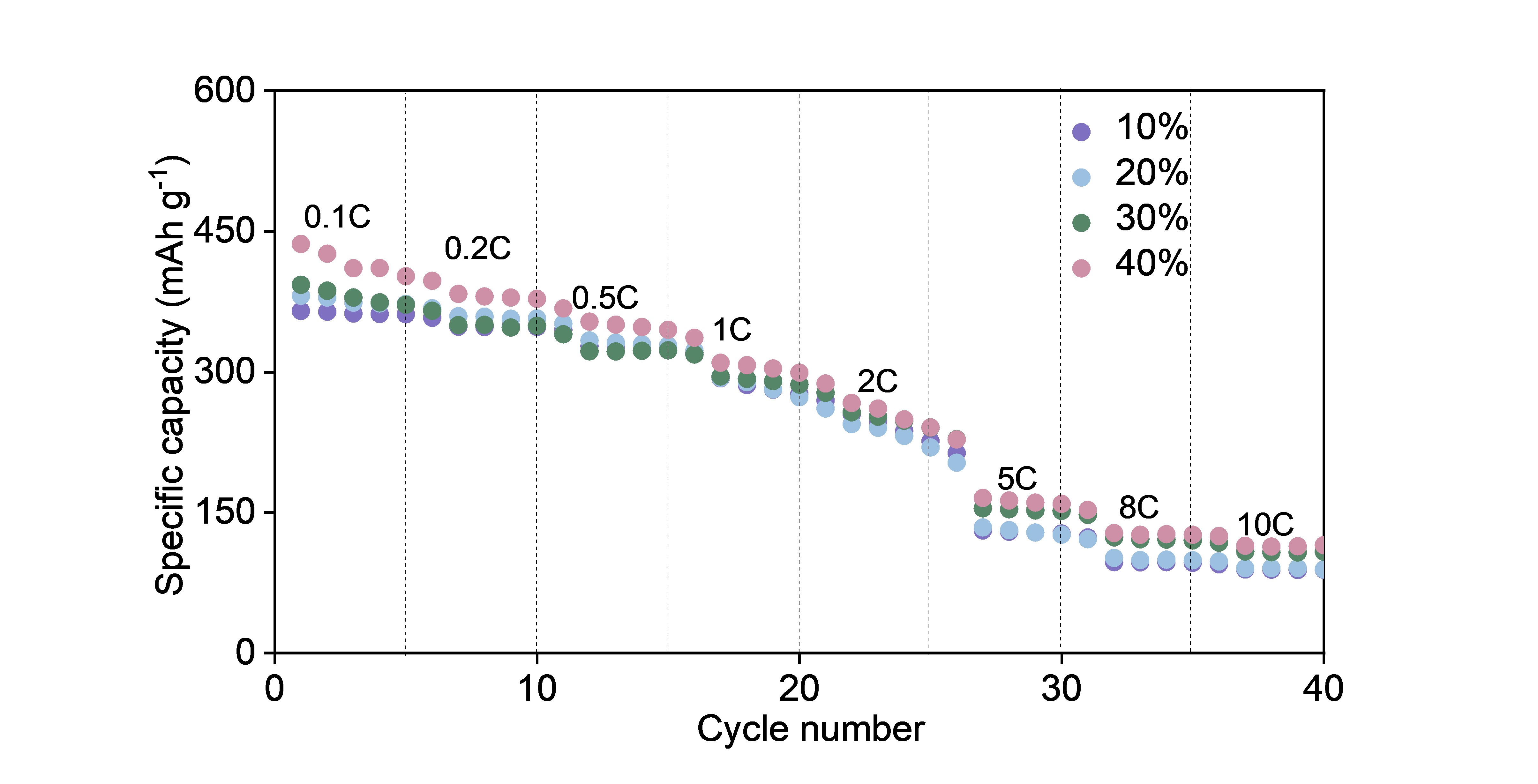


Figure S15. Rate performance of graphite/HC-GF blended anodes with various mass ratios.


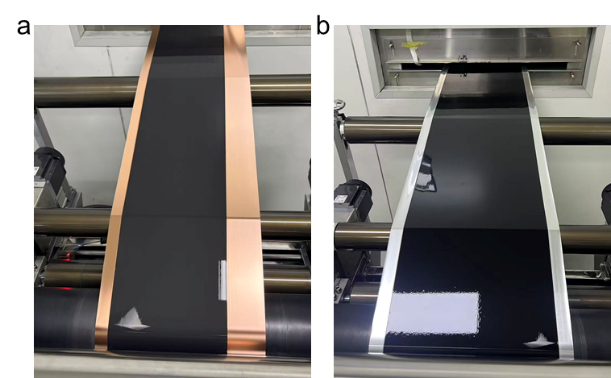


Figure S16. Digital photographs of (a) Graphite+20%(HC-GF) anode and (b) LFP cathode coatings.


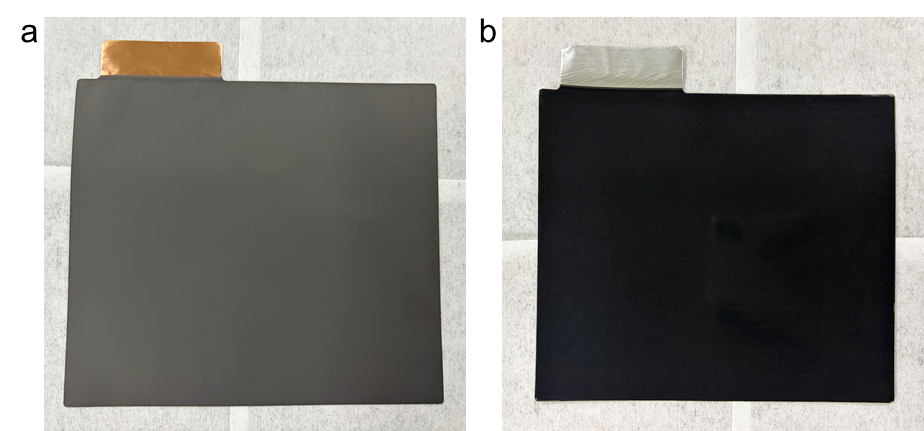


Figure S17. Digital photographs of (a) Graphite+20%(HC-GF) anode and (b) LFP cathode electrode sheets.


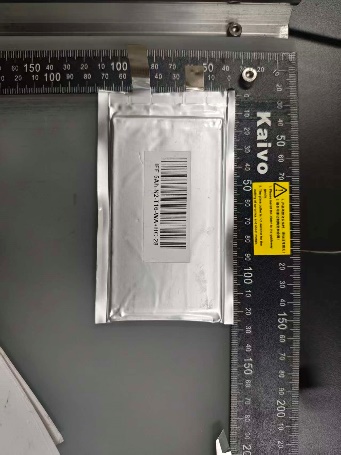


Figure S18. Digital photograph of the LFP//Graphite+20%(HC-GF) pouch battery.


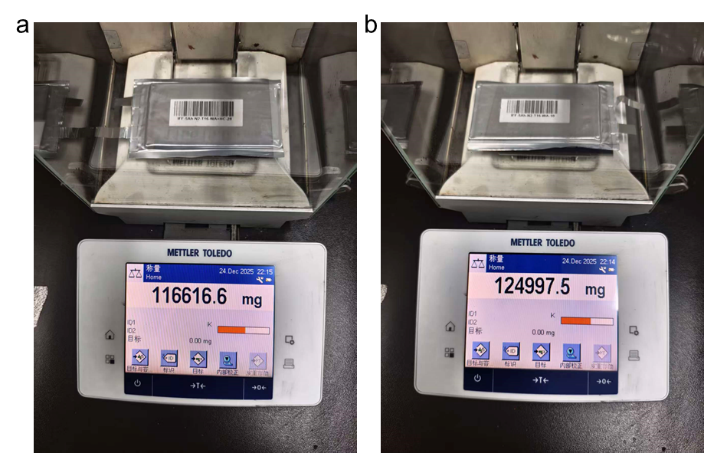


Figure S19. Digital photographs of the mass measurement for (a) LFP//Graphite+20%(HC-GF) and (b) LFP//Graphite pouch batteries.


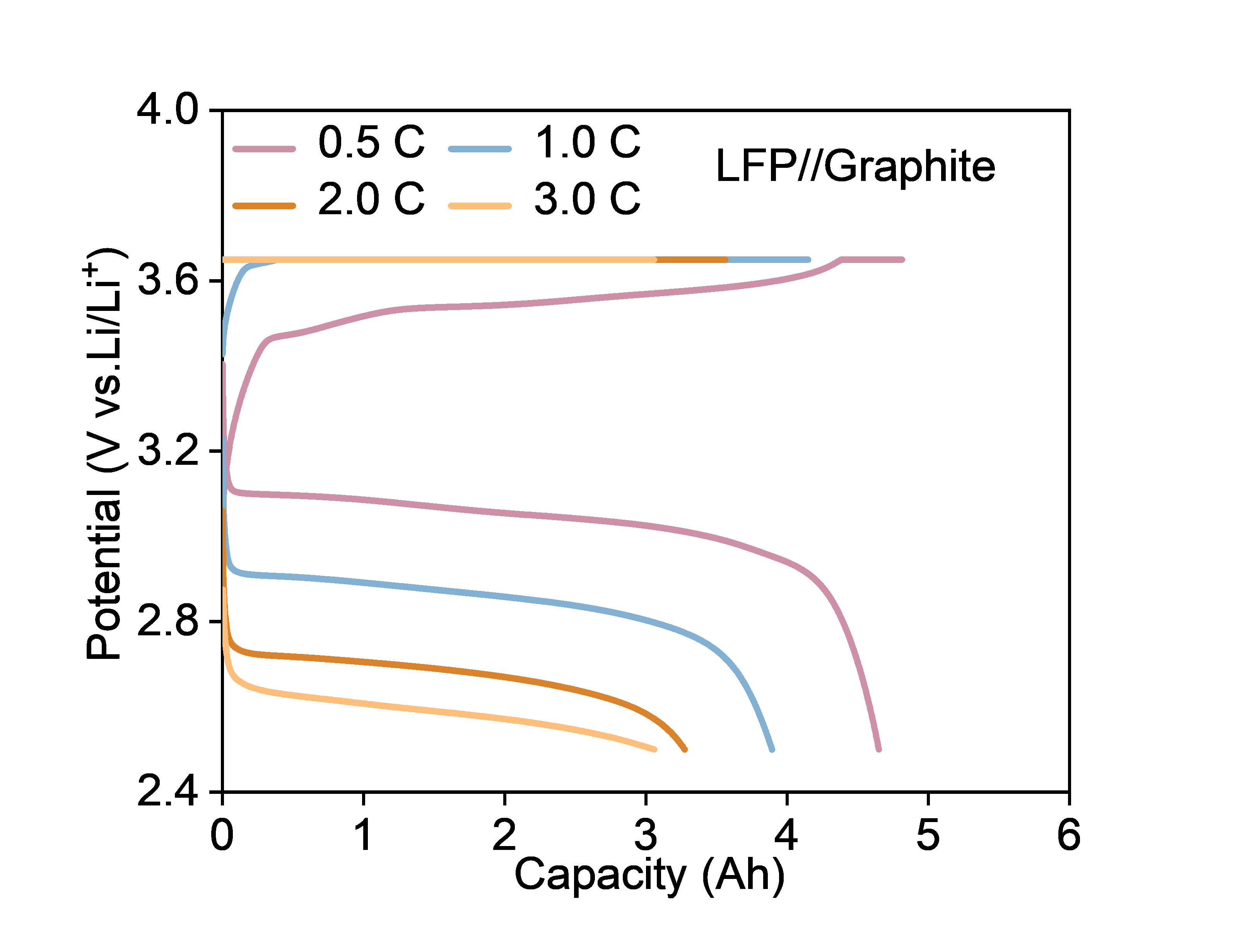


Figure S20. Charge-discharge profiles of the 5 Ah LFP//Graphite pouch battery at various rates.


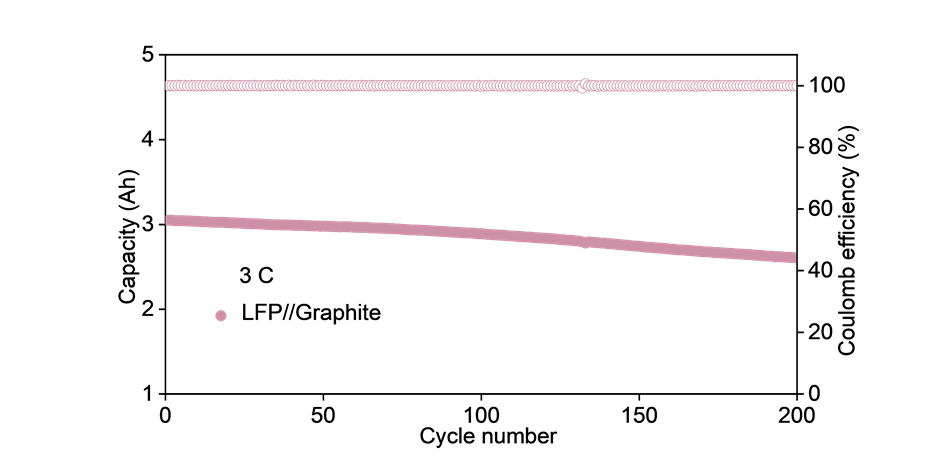


Figure S21. Cycling stability of the 5 Ah LFP//Graphite pouch battery at various rates.
